# Regulation of FADS2 transcription by SREBP-1 and PPAR-α influences LC-PUFA biosynthesis in fish

**DOI:** 10.1038/srep40024

**Published:** 2017-01-09

**Authors:** Xiaojing Dong, Peng Tan, Zuonan Cai, Hanlin Xu, Jingqi Li, Wei Ren, Houguo Xu, Rantao Zuo, Jianfeng Zhou, Kangsen Mai, Qinghui Ai

**Affiliations:** 1 Key Laboratory of Aquaculture Nutrition and Feed (Ministry of Agriculture) and Key Laboratory of Mariculture (Ministry of Education), Ocean University of China, 5 Yushan Road, Qingdao, Shandong 266003, People’s Republic of China; 2 Laboratory for Marine Fisheries and Aquaculture, Qingdao National Laboratory for Marine Science and Technology, Qingdao, Shandong 266003, People’s Republic of China; 3 Key Laboratory of Marine Drugs, Ministry of Education, Ocean University of China, 5 Yushan Road, Qingdao, Shandong 266003, People’s Republic of China

## Abstract

The present study was conducted to explore the mechanisms leading to differences among fishes in the ability to biosynthesize long-chain polyunsaturated fatty acids (LC-PUFAs). Replacement of fish oil with vegetable oil caused varied degrees of increase in 18-carbon fatty acid content and decrease in n-3 LC-PUFA content in the muscle and liver of rainbow trout (*Oncorhynchus mykiss*), Japanese seabass (*Lateolabrax japonicus*) and large yellow croaker (*Larimichthys crocea*), suggesting that these fishes have differing abilities to biosynthesize LC-PUFAs. Fish oil replacement also led to significantly up-regulated expression of FADS2 and SREBP-1 but different responses of the two PPAR-α homologues in the livers of these three fishes. An *in vitro* experiment indicated that the basic transcription activity of the FADS2 promoter was significantly higher in rainbow trout than in Japanese seabass or large yellow croaker, which was consistent with their LC-PUFA biosynthetic abilities. In addition, SREBP-1 and PPAR-α up-regulated FADS2 promoter activity. These regulatory effects varied considerably between SREBP-1 and PPAR-α, as well as among the three fishes. Taken together, the differences in regulatory activities of the two transcription factors targeting FADS2 may be responsible for the different LC-PUFA biosynthetic abilities in these three fishes that have adapted to different ambient salinity.

Decreasing global availability, coupled with the high cost of fish oil, has forced the aquaculture industry to investigate possible alternative sources of dietary lipids. Vegetable oils stand out as the most likely candidates for partial substitutes for fish oils in fish feeds because of their lower price and higher levels of production. Some vegetable oils, such as soybean oil and linseed oil, are considered good alternative lipid sources for salmonids and freshwater fish^1^,^2^,^3^. Although replacing fish oil with vegetable oil generally does not affect the overall health and growth of the fish, most studies have shown that the fish possess reduced levels of long-chain polyunsaturated fatty acids (LC-PUFAs), particularly of DHA and EPA, which are indispensable for their growth and nutrition^4^,^5^,^6^,^7^.

In comparison with freshwater fish, marine fish species generally lack the ability to synthesize LC-PUFAs from their 18-carbon precursor fatty acids^8^,^9^. However, euryhaline fish commonly show varying levels of capacity to synthesize LC-PUFAs, depending on the ambient salinity^10^,^11^,^12^. Because FADS2 has been shown to catalyse the first limiting step in the LC-PUFA biosynthesis pathway in mammals, special attention has been given to characterization of the FADS2 product^13^,^14^. Thus, FADS2 has been cloned, and its nutritional regulation has been widely investigated in many fish species^15^,^16^,^17^,^18^,^19^,^20^,^21^,^22^,^23^,^24^,^25^. Although the expression level of FADS2 generally indicates the capacity for LC-PUFA synthesis in fish^16^,^26^, the underlying mechanisms by which FADS2 expression is regulated have rarely been reported.

Two transcription factors, SREBP and PPAR, are involved in the regulation of fatty acid biosynthesis in mammals^27^,^28^,^29^,^30^,^31^,^32^,^33^, by binding to sterol regulatory elements (SREs)^27^,^34^ and peroxisome proliferator response elements (PPREs)^35^, respectively. In fish, previous studies have shown that SREBP-1 is related to fatty acid metabolism^36^,^37^ and that the gene expression of SREBP-1 and PPAR-α can be regulated by dietary fatty acids^33^. However, it remains unclear whether these two factors are involved in fatty acid biosynthesis by targeting FADS2.

In the present study, a 70 d feeding experiment was conducted on rainbow trout, Japanese seabass and large yellow croaker to comprehensively compare the effects of different levels of vegetable oil substitution on tissue fatty acid composition and on the expression of genes related to LC-PUFA biosynthesis (FADS2, SREBP-1 and PPAR-α). Then, an *in vitro* experiment was conducted to investigate the activity of SREBP-1 and PPAR-α in regulating the expression of the FADS2 gene promoter.

## Results

### Growth and survival performance

In rainbow trout, partial or total replacement of fish oil with vegetable oil had no significant effects on SGR, FER, SR and FI compared with the control group. In Japanese seabass, however, increasing the level at which fish oil was replaced with vegetable oil significantly decreased the SGR, FER, FI and SR (*P* < 0.05), although the survival rate showed no significant difference between the FV and VO groups. For large yellow croaker, SGR, FER and SR were significantly reduced by fish oil substitution; however, no significant difference was observed between the 50% and 100% substitutions. Interestingly, fish oil replacement showed no influence on feed intake in large yellow croaker, which was also true with rainbow trout (Table 1).

### Fatty acid compositions in the liver and muscle

Vegetable oil substitution caused an increase in 18-carbon fatty acids, such as C18:3n-3 and C18:2n-6, in both the livers and muscles of all three fishes, especially in the liver of large yellow croaker, in which the increases reached 4.5-fold (from 1.49 mg/g to 6.74 mg/g) and 5-fold (from 6.8 mg/g to 34.64 mg/g) in the 50% and 100% replacement groups, respectively. The fish fed 50% or 100% vegetable oil showed significantly lower contents of n-3 LC-PUFAs in the muscle and liver than did the fish fed fish oil. The decrease was greatest in large yellow croaker; for example, C20:5n-3 content in muscle decreased from 2.58 mg/g (FO) to 0.84 mg/g (FV) and 0.15 mg/g (VO). However, no significant difference was observed in the C22:6n-3 content of the muscle between rainbow trout fed 50% vegetable oil and those fed 100% fish oil (Tables 2 and 3).

### Cloning and characterization of the promoter and cDNA

**Figure 1:**
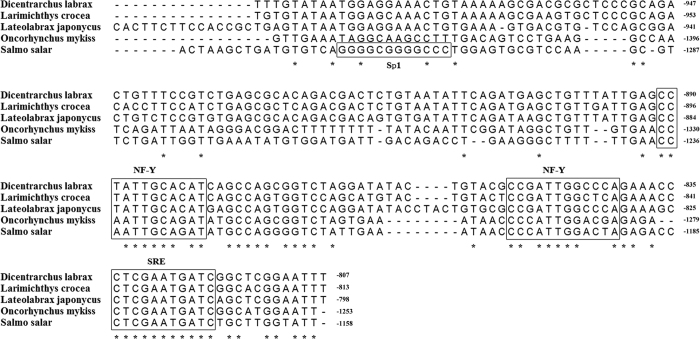
Alignment of FADS2 promoter fragments among fish. The numbers indicate sequence position relative to the translation initiation site (ATG). Binding sites are indicated based on previous work from Zheng^36^ and Geay^56^.

The upstream sequences adjacent to the translation start codon of FADS2 in rainbow trout and large yellow croaker were cloned by genome walking, and the fragments were 1479 bp and 996 bp in length, respectively. The promoter sequence of Japanese seabass was published in our previous study^24^. Alignment analysis showed that the FADS2 promoters of rainbow trout, Japanese seabass and large yellow croaker possess transcription factor binding elements including those for nuclear factor Y (NF-Y) and SRE, as in European sea bass and Atlantic salmon, but they lack Sp1 elements (Fig. 1).

The full lengths of the putative SREBP-1 cDNAs from rainbow trout (GenBank accession number: KP342261) and large yellow croaker (GenBank accession number: KP342262) were 4220 bp and 3750 bp in length, respectively, each encoding a protein with high identity to mammalian SREBP-1. The deduced amino acid sequence displayed the typical structure of SREBP, i.e., the bHLH-Zip domain. Alignment analysis of the N-terminal protein sequence showed that SREBP-1 in fish was more similar in length to human SREBP-1a than to SREBP-1c (Fig. S1). The phylogenetic tree showed that fish species clustered together and formed a sister group to the branch for mammals and chicken (Fig. S2).

**Figure 2:**
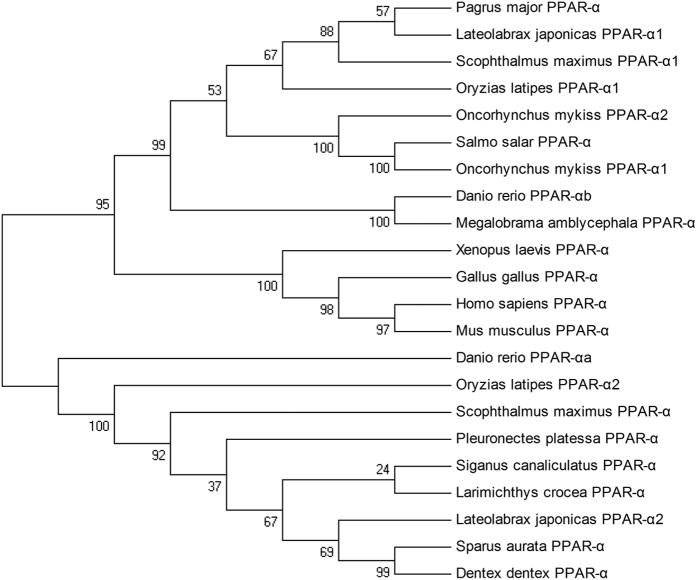
Phylogenetic relationship of the amino acid sequences of PPAR-α from vertebrates and invertebrates.

In the present study, a new putative PPAR-α cDNA (GenBank accession number: KP342260, named PPAR-α2 here) from rainbow trout was cloned that differed from the previously reported one (GenBank accession number: NM_001197211, named PPAR-α1 here) (Fig. S3). The deduced protein sequences of PPAR-α2 from rainbow trout displayed the typical structure of PPAR, i.e., contained a C4-type zinc finger and a ligand-binding domain (Fig. S3). The phylogenetic tree of PPAR-α was obviously divided into two branches, each harbouring one PPAR-α isoform for the fish possessing two sequences, except for rainbow trout (Fig. 2).

### Gene expression in response to dietary fatty acids

The FADS2 transcript showed significantly higher expression in the livers of the fish fed vegetable oil than in those of the fish fed fish oil. The SREBP-1 transcript also showed higher expression in the fish fed vegetable oil compared with the fish fed fish oil. For PPAR-α, replacement of fish oil with vegetable oil significantly up-regulated its expression in the liver of rainbow trout but significantly down-regulated its expression in the liver of Japanese seabass. In large yellow croaker, however, no significant differences between groups were detected. These results showed that among these fishes, the levels of transcription of SREBP-1 and PPAR-α in the liver respond differently to dietary vegetable oil.

### Promoter activity in cells

To determine the promoter activity of FADS2 and the roles of SREBP-1 and PPAR-α in regulating FADS2 promoter activity in the three fishes, HEK293T cells were co-transfected with the FADS2- promoter luciferase reporter plasmid and the SREBP-1 or PPAR-α expression plasmid, using PGL3-Basic and PCS2+ as controls in the dual-luciferase reporter assay.

**Figure 3:**
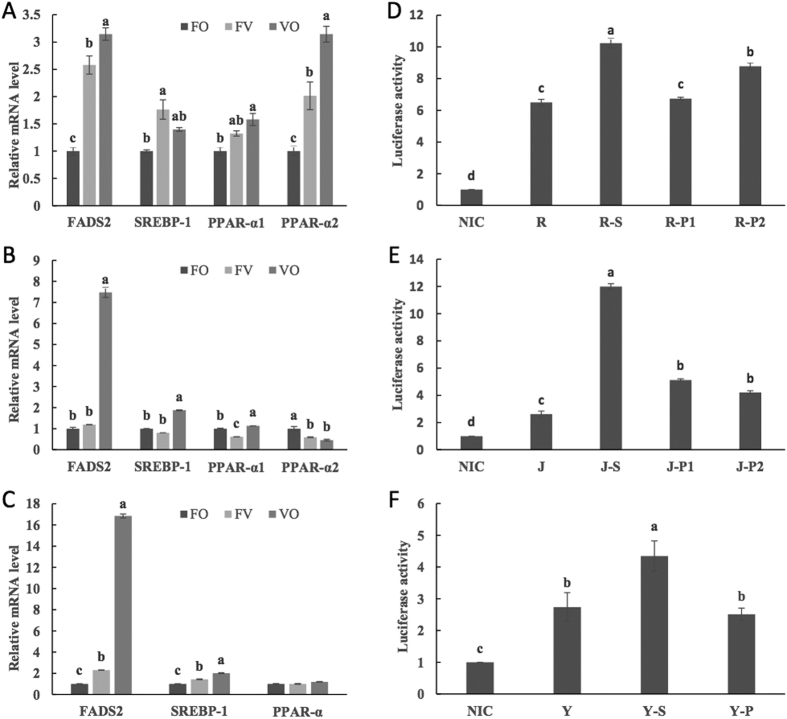
Relative gene transcription levels in the livers of experimental fishes (**A–C**) and dual-luciferase detection results (**D–F**). Gene transcription levels and luciferase activity are presented as the mean ± SEM (n = 3). The plasmid transfection groups are as follows: NIC: PGL3-Basic + PCS2+ + PRL-CMV; R: PGL-RF + PCS2+ + PRL-CMV; R-S: PGL-RF + PCS-RS + PRL-CMV; R-P1: PGL-RF + PCS-RP1 + PRL-CMV; R-P2: PGL-FR + PCS-RP2 + PRL-CMV; J: PGL-JF + PCS2+ + PRL-CMV; J-S: PGL-JF + PCS-JS + PRL-CMV; J-P1: PGL-JF + PCS-JP1 + PRL-CMV; J-P2: PGL-JF + PCS-JP2 + PRL-CMV; Y: PGL-YF + PCS2+ + PRL-CMV; Y-S: PGL-YF + PCS-YS + PRL-CMV; Y-P: PGL-YF + PCS-YP + PRL-CMV. Different letters above the bars denote significant differences between diet groups at the *P *< 0.05 level.

In HEK293T cells, the promoter activity of FADS2 in rainbow trout was significantly higher than those in Japanese seabass and large yellow croaker, whereas no significant difference was observed between Japanese seabass and large yellow croaker (groups R, J and Y) (Fig. 3). The transcription factor SREBP-1 up-regulated the promoter activity of FADS2 by 1.58-fold, 4.57-fold and 1.59-fold in rainbow trout, Japanese seabass and large yellow croaker, respectively. The transcription factor PPAR-α up-regulated the promoter activity of FADS2 in rainbow trout and Japanese seabass but not in large yellow croaker. Interestingly, only the newly cloned PPAR-α gene of rainbow trout, i.e., PPAR-α2, showed regulatory activity on the promoter of FADS2 in HEK293T cells (Fig. 3).

## Discussion

The vegetable oils, soybean oil and linseed oil, are relatively rich in 18-carbon fatty acids but have low levels of LC-PUFAs in comparison with fish oil. LC-PUFAs are essential fatty acids for marine fish, and they may lack or have less ability to transform 18-carbon fatty acids into LC-PUFAs. Thus, replacement of fish oil with vegetable oil can negatively affect the growth and survival of marine fishes. The results of this study showed that full replacement did not affect the SGR, FER, FI and SR in rainbow trout (freshwater) but substantially reduced the growth and survival in Japanese seabass (euryhaline) and large yellow croaker (marine). Correspondingly, fishes fed vegetable oil (50% or 100%) had high levels of C18:3n-3 and C18:2n-6 but low levels of n-3 LC-PUFAs in their muscles and liver, and this was especially pronounced in the large yellow croaker. Previous studies have demonstrated that marine fish species show less ability to synthesize LC-PUFAs from C18:3n-3 and C18:2n-6^8^,^9^,^22^,^38^,^39^,^40^. However, the mechanisms involved in the dietary regulation of LC-PUFA synthesis have rarely been studied in fish to date. FADS2 is a key enzyme catalysing the first rate-limiting step in the biosynthesis of LC-PUFA from C18:3n-3 and C18:2n-6 and thus is commonly used as an indicator of LC-PUFA biosynthesis^16^,^24^,^26^, but the underlying mechanism in fish has been poorly understood.

Both SREBP-1 and PPAR-α are major regulators of fatty acid metabolic genes including those involved in LC-PUFA synthesis. Two forms of mammalian SREBP-1 have been characterized, SREBP-1a and -1c. However, to date, only a single form of the SREBP-1 gene has been characterized in fish^33^,^37^,^41^, with no exception for the three fishes investigated in the present study. The alignment analysis of the deduced amino acid sequences showed that the SREBP-1 genes from fish were more similar to human SREBP-1a than to human SREBP-1c, indicating that fish SREBP-1 genes likely only possess functions similar to those of human SREBP-1a transcripts^33^,^37^. Unlike in mammals, two forms of the PPAR-α gene were characterized in rainbow trout, which was consistent with Japanese seabass, fugu, zebrafish, Japanese medaka, turbot and grass carp^33^,^42^,^43^. However, only a single orthologue was found in large yellow croaker, as observed in the olive flounder^44^. The phylogenetic analysis revealed that except in rainbow trout, fish PPAR-α1 and PPAR-α2 were on different branches. It has been hypothesized that gene duplication might have occurred during the evolution of PPAR-α, resulting in two orthologues with divergent functions in some fishes^33^,^45^.

Although not always attaining statistical significance, several studies have shown that replacing fish oil with vegetable oil consistently results in increased FADS2 transcription in fish^1^,^26^,^39^,^46^,^47^,^48^,^49^,^50^,^51^,^52^,^53^. In the present study, the transcript level of FADS2 was significantly higher in the livers of the fish fed vegetable oil than in those of the fish fed fish oil. Moreover, replacement with vegetable oil significantly up-regulated the transcription level of SREBP-1 in most cases, consistent with previous studies^33^,^54^. Interestingly, in Japanese seabass fed vegetable oil, the two isotypes of PPAR-α showed different responses, which may indicate that functional differentiation has occurred since the gene duplication^33^,^55^. In addition, the transcription level of PPAR-α was not significantly influenced by the dietary fatty acids in large yellow croaker. Given that PPAR-α plays important regulatory roles in LC-PUFA biosynthesis in mammals, the different responses of PPAR-α to dietary fatty acids might be responsible for the different LC-PUFA biosynthesis abilities among fishes.

Previous studies suggested that the lack of binding sites for the transcription factor Sp1 may explain the lower activity of the FADS2 promoter in Atlantic cod than in Atlantic salmon^36^,^56^. Although the FADS2 promoter of rainbow trout showed significantly higher activity than those of the Japanese seabass and large yellow croaker in HEK293T cells, no Sp1 binding site was identified in the promoter region of rainbow trout. Thus, there must be other transcription factors regulating the transcription activity of FADS2. In mammals, transcription of FADS2 is dually regulated by SREBP-1 and PPAR-α, two reciprocal transcription factors for fatty acid metabolism^27^,^57^,^58^. *In vitro* analysis showed that as in mammals, the transcription factor SREBP-1 up-regulated the promoter activity of FADS2 to varying degrees in the three fishes. Moreover, both PPAR-α1 and PPAR-α2 were demonstrated to up-regulate the promoter activity of FADS2 in Japanese seabass, but no such regulatory activity was detected in large yellow croaker. In contrast, in rainbow trout, only PPAR-α2 showed regulatory activity, although both PPAR-α1 and PPAR-α2 were on the same evolutionary branch. Given that the salmonids are still in the process of reverting to a stable diploid state after a genome duplication event^59^, the structural similarity and functional differentiation of the two isotypes of PPAR-α in rainbow trout might suggest that the duplicated PPAR-α gene is still in the process of accumulating mutations.

In conclusion, the present study suggests that two transcription factors, SREBP-1 and PPAR-α, are involved in fatty acid biosynthesis via regulating FADS2 and that the differences in their regulatory activity may be responsible for differential LC-PUFA biosynthesis abilities among fishes that have adapted to different ambient salinity.

## Materials and Methods

### Ethics statement

The present experimental procedures were carried out in strict accordance with the recommendations in the Guide for the Use of Experimental Animals of Ocean University of China. All animal care and use procedures were approved by the Institutional Animal Care and Use Committee of Ocean University of China (Permit Number: 20001001). Before handling and sacrifice, experimental fish were anesthetized with MS-222 (250 mg/L, Sigma), and all efforts were made to minimize suffering.

### Feeding trial

Three isoprotein (41% crude protein) and isolipidic (12% crude lipid) diets were formulated to contain graded levels of vegetable oil blend (0, 50 and 100%) by supplementation of soybean oil and linseed oil (Tables 4 and 5). The three artificial diets were designated FO (control), FV and VO.

Rainbow trout was obtained from a commercial farm in Weifang, Shandong, China. Fish similar in size were randomly sorted into tanks that were supplied with a continuous flow of freshwater with continuous aeration. During the entire experiment, the water temperature was kept at 18 ± 3 °C, with dissolved oxygen at approximately 7–8 mg/L. Large yellow croaker and Japanese seabass were purchased from a local fish farm in Xiangshan Bay, Zhejiang, China. Prior to the beginning of the experiment, fish of similar sizes were randomly grouped and reared in floating sea cages for one week. During the experiment, all environmental parameters were the same as in the practical cultivation environment; i.e., the temperature was 24–29 °C, the salinity was 29–32‰, and the dissolved oxygen was approximately 6–7 mg/L.

Each diet was randomly assigned to triplicate tanks or cages. To prevent wasting of dietary pellets, the fish were slowly hand-fed little by little until apparent satiation on the basis of visual observation. The fish were fed twice daily, at 08:00 and 17:00, for 10 weeks.

### Assay of fatty acid composition

The fatty acid composition was determined via gas chromatography–mass spectrometry (GC–MS) using a Thermo TRACE 1310 GC-ISQ QD MS mass spectrometer equipped with an Agilent 7890AGC-5975CMS gas chromatograph.

### RNA extraction, cDNA synthesis and quantitative real-time PCR

Total RNA was extracted from the liver using RNAiso Plus (TaKaRa Bio, Dalian, China) according to the manufacturer’s protocol. Then, 1 μg of total RNA was subjected to PrimeScript^®^RT reagent Kit with gDNA Eraser (TaKaRa Bio) in a 20 μl volume for reverse transcription and DNA removal.

Real-time PCR was conducted in a quantitative thermal cycler (Eppendorf, Hamburg, Germany). The amplification was performed in a total volume of 25 ml, containing 1 μl of each primer (10 mM), 1 μl of the diluted first strand cDNA product, 12.5 μl of 2 × SYBR Premix Ex Taq II (TaKaRa Bio) and 9.5 μl of sterilized double-distilled water. The primer sequences for β-actin, FADS2, SREBP-1, PPAR-α1 and PPAR-α2 were designed using Primer Premier 5.0 (Table 6). Each sample was run in triplicate. For each run, PCR controls were assayed and PCR efficiency was measured by the slope of a standard curve using serial dilutions of cDNA. PCR amplification efficiency values ranged between 0.90 and 1.10 in all cases. The gene expression levels of putative FADS2, SREBP-1, PPAR-α1, and PPAR-α2 were determined using the 2^−ΔΔCT^ method^60^.

### Luciferase Reporter Assay

The genome-walking experiment was conducted to isolate the promoter sequences of FADS2 of rainbow trout, Japanese seabass and large yellow croaker. Briefly, genomic DNAs were extracted from the liver tissue using the SQ Tissue DNA Kit (Omega Bio-Tek, Norcross, America), and specific reverse primers were designed for each species (Table S1) based on the sequences of rainbow trout (NM_001124287.1), Japanese seabass (JX678842.1) and large yellow croaker (NM_001303363). Three rounds of genome walking were conducted for each species, using the forward primer AP4 supplied in the kit (Takara Bio) according to the manufacturer’s instructions.

For cloning the cDNAs of *SREBP* and *PPAR-α*, degenerate primers were designed based on highly conserved regions of the genes from other fishes available in the GenBank database. Then, gene-specific primers were further designed (Table S2) to clone the 3′ and 5′ ends by rapid amplification of cDNA ends (RACE) technology using the SMARTer^TM^ RACE cDNA Amplification Kit (Clontech, Mountain View, America). PCR products were cloned into the pEASY-T1 simple cloning vector (TransGen, Beijing, China) and sequenced by Sangon Biotech (Shanghai, China).

Similar searches of the cloned cDNA sequences were conducted using Blastn against the NCBI database (www.ncbi.nlm.nih.gov/blast/). Multiple-sequence alignment was carried out using ClustalW, followed by the construction of phylogenetic trees by using the neighbour-joining method in MEGA version 4.0.

For functional analysis, coding sequences of SREBP-1 and PPAR-α from rainbow trout, Japanese seabass and large yellow croaker were sub-cloned into the PCS2+ expression vectors, named PCS-RS, PCS-RP1, PCS-RP2, PCS-JS, PCS-JP1, PCS-JP2, PCS-YS, and PCS-YP. The FADS2 promoters from the three fishes were sub-cloned into the PGL3-Basic vector (Promega, Beijing, China), which does not include a promoter but contains a luciferase reporter gene, and named PGL-RF, PGL-JF and PGL-YF. All recombinant constructs were sequenced to verify the orientation before *in vitro* experiments in HEK293T cells. (Table S3).

HEK293T cells were maintained in DMEM supplied with 10% foetal bovine serum. Cells were seeded into 24-well plates. When they reached 60–70% confluence, the plasmids were transfected into duplicate wells using the Lipofectamine 2000 system (Invitrogen, Carlsbad, America). For each experiment, 600 ng of PCS plasmid, 200 ng of PGL3 plasmid, and 20 ng of the Renilla luciferase reporter plasmid PRL-CMV (Promega) were mixed and transfected at 26 °C.

The cells were cultured for 6 hours, followed by replacement of the transfection medium with fresh medium and further incubation. Luciferase activities were measured 24 h after the transfection using a dual-luciferase assay kit (Promega). The relative luciferase activities of the promoters were normalized to the control reporter. The final data were from three independent experiments, and each experiment was performed in triplicate.

### Calculations and statistical methods


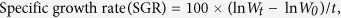



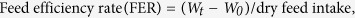



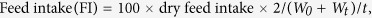



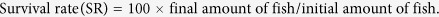


*W*_*t*_ and *W*_*0*_ are the final and initial body weights, respectively, and *t* is the duration of the experiment in days.

Differences in the fatty acid composition and gene expression due to the different diets were determined by one-way ANOVA using SPSS 18.0 (SPSS Inc., Chicago, America), and Duncan’s multiple range test was used to assess differences among groups. If unequal variance was determined using Levene’s test, data were log-transformed before statistical analysis. Data are expressed as the means ± SEM, and *P* values of less than 0.05 were considered statistically significant.

## Additional Information

**How to cite this article**: Dong, X. *et al*. Regulation of FADS2 transcription by SREBP-1 and PPAR-α influences LC-PUFA biosynthesis in fish. *Sci. Rep.* **7**, 40024; doi: 10.1038/srep40024 (2017).

**Publisher's note:** Springer Nature remains neutral with regard to jurisdictional claims in published maps and institutional affiliations.

## Supplementary Material

Supplementary Information

## Figures and Tables

**Table 1:** Growth performance and survival rates of three fishes fed experimental diets with vegetable oil instead of fish oil (Mean ± SEM)*.

	RFO^1^	RFV^2^	RVO^3^	JFO^4^	JFV^5^	JVO^6^	LFO^7^	LFV^8^	LVO^9^
IBW^10^ (g)	11.36 ± 0.34	11.24 ± 0.31	11.14 ± 0.32	18.66 ± 0.36	18.55 ± 0.28	18.59 ± 1.07	9.00 ± 0.41	9.09 ± 0.23	8.69 ± 0.46
FBW^11^ (g)	47.68 ± 1.42	50.95 ± 2.27	48.36 ± 1.90	86.83 ± 1.76^a^	76.90 ± 1.56^b^	52.66 ± 1.15^c^	31.51 ± 1.19^a^	27.64 ± 0.81^b^	23.69 ± 0.77^c^
SGR^12^ (g d^−1^)	1.95 ± 0.04	2.00 ± 0.07	1.95 ± 0.06	2.19 ± 0.02^a^	2.01 ± ± 0.02^b^	1.49 ± 0.03^c^	1.79 ± 0.08^a^	1.59 ± 0.05^ab^	1.43 ± 0.05^b^
FER^13^	0.68 ± 0.02	0.72 ± 0.02	0.69 ± 0.01	0.90 ± 0.01^a^	0.75 ± 0.02^b^	0.46 ± 0.01^c^	0.64 ± 0.03^a^	0.54 ± 0.02^b^	0.52 ± 0.03^b^
FI^14^ (% d^−1^)	2.51 ± 0.05	2.41 ± 0.02	2.44 ± 0.03	1.84 ± 0.01^a^	1.74 ± 0.01^b^	1.37 ± 0.02^c^	2.52 ± 0.04	2.64 ± 0.04	2.56 ± 0.06
SR^15^ (%)	98.67 ± 1.33	98.67 ± 1.33	94.67 ± 1.33	78.89 ± 1.11^a^	63.33 ± 1.92^b^	65.56 ± 2.22^b^	88.33 ± 0.96^a^	67.22 ± 1.26^b^	62.78 ± 1.82^b^

^*^The statistical analysis was conducted in each fish species.

^1^RFO: 100% Fish oil as lipid source (control) in rainbow trout.

^2^RFV: Vegetable oil blend (linseed oil: soya bean oil = 1:1) replacing 50% of fish oil in rainbow trout.

^3^RVO: 100% Vegetable oil blend as lipid source in rainbow trout.

^4^JFO: 100% Fish oil as lipid source (control) in Japanese seabass.

^5^JFV: Vegetable oil blend (linseed oil: soya bean oil = 1:1) replacing 50% of fish oil in Japanese seabass.

^6^JVO: 100% Vegetable oil blend as lipid source in Japanese seabass.

^7^LFO: 100% Fish oil as lipid source (control) in large yellow croaker.

^8^LFV: Vegetable oil blend (linseed oil: soya bean oil = 1:1) replacing 50% of fish oil in large yellow croaker.

^9^LVO: 100% Vegetable oil blend as lipid source in large yellow croaker.

^10^IBW: Initial body weight.

^11^FBW: Final body weight.

^12^SGR = 100 × (ln *W*_*t*_ − ln *W*_*0*_)/*t*.

^13^FER = (*W*_*t*_ − *W*_*0*_)/dry feed intake.

^14^FI = 100 × dry feed intake × 2/(*W*_*0*_ + *W*_*t*_)/t.

^15^SR = 100 × final amount of fish/initial amount of fish.

**Table 2:** Muscle fatty acid contents of three fishes fed the experimental diets with vegetable oil instead of fish oil (mg/g, Mean ± SEM)*.

Fatty acid	RFO^1^	RFV^2^	RVO^3^	JFO^4^	JFV^5^	JVO^6^	LFO^7^	LFV^8^	LVO^9^
C14:0	1.09 ± 0.00^a^	0.75 ± 0.00^b^	0.75 ± 0.02^b^	1.09 ± 0.00^a^	0.79 ± 0.01^c^	0.86 ± 0.02^b^	1.76 ± 0.08^a^	1.20 ± 0.03^b^	0.78 ± 0.01^c^
C16:0	4.35 ± 0.00^a^	3.41 ± 0.18^b^	3.21 ± 0.09^b^	2.95 ± 0.18^a^	1.90 ± 0.07^b^	2.22 ± 0.07^b^	7.17 ± 0.43^a^	6.04 ± 0.29^a^	4.24 ± ± 0.38^b^
C18:0	1.40 ± 0.01	1.36 ± 0.12	1.59 ± 0.00	1.50 ± ± 0.06^a^	1.08 ± 0.03^b^	1.44 ± 0.04^a^	2.98 ± 0.09	3.01 ± 0.31	2.48 ± 0.22
∑SFA^10^	6.84 ± 0.02^a^	5.53 ± 0.30^b^	5.55 ± 0.09^b^	5.54 ± 0.25^a^	3.78 ± 0.10^c^	4.52 ± 0.11^b^	11.90 ± 0.51^a^	10.25 ± 0.61^a^	7.51 ± 0.59^b^
C16:1	0.96 ± 0.09^a^	0.49 ± 0.04^b^	0.29 ± 0.01^b^	0.41 ± 0.06^a^	0.24 ± 0.03^b^	0.15 ± 0.01^b^	2.51 ± 0.53^a^	1.33 ± 0.19^ab^	0.38 ± 0.05^b^
C18:1	3.40 ± 0.43^ab^	4.48 ± 0.28^a^	2.30 ± 0.10^b^	1.99 ± 0.39	1.76 ± 0.18	2.03 ± 0.09	8.66 ± 0.53^a^	7.97 ± 0.68^a^	4.64 ± 0.71^b^
∑MUFA^11^	4.36 ± 0.52^a^	4.97 ± 0.26^a^	2.59 ± 0.10^b^	2.40 ± 0.44	2.00 ± 0.18	2.18 ± 0.10	11.18 ± 0.64^a^	9.31 ± 0.86^a^	5.01 ± 0.71^b^
C18:2n-6	2.58 ± 0.57^b^	5.39 ± 0.44^a^	5.61 ± 0.31^a^	1.89 ± 0.43^b^	2.44 ± 0.38^b^	4.46 ± 0.27^a^	7.41 ± 0.04^b^	11.85 ± 0.46^a^	14.55 ± 1.24^a^
C20:4n-6	0.20 ± 0.01^a^	0.14 ± 0.00^b^	0.11 ± 0.01^b^	0.22 ± 0.02^a^	0.17 ± 0.01^ab^	0.14 ± 0.01^b^	0.28 ± 0.04^a^	0.16 ± 0.01^b^	0.10 ± 0.00^b^
∑n-6 PUFA^12^	2.78 ± 0.59^b^	5.53 ± 0.44^a^	2.72 ± 0.31^b^	2.11 ± 0.40^b^	2.62 ± 0.38^b^	4.60 ± 0.27^a^	7.70 ± 0.04^b^	12.01 ± 0.47^a^	14.65 ± 1.24^a^
C18:3n-3	0.30 ± 0.03^b^	1.14 ± 0.26^a^	0.95 ± 0.09^a^	0.27 ± 0.06^c^	0.66 ± 0.03^b^	1.21 ± ± 0.11^a^	0.54 ± 0.22^b^	4.59 ± 0.59^a^	5.76 ± 1.51^a^
C20:5n-3	0.82 ± 0.10^a^	0.47 ± 0.05^b^	0.12 ± 0.00^c^	0.64 ± 0.05^a^	0.44 ± 0.04^b^	0.18 ± 0.01^c^	2.58 ± 0.26^a^	0.84 ± 0.06^b^	0.15 ± 0.02^c^
C22:6n-3	5.24 ± 0.32^a^	4.59 ± 0.21^a^	1.10 ± 0.10^b^	2.28 ± 0.06^a^	1.65 ± 0.11^b^	0.82 ± 0.06^c^	5.25 ± 0.40^a^	2.05 ± 0.21^b^	0.64 ± 0.11^c^
∑n-3 PUFA^13^	6.36 ± 0.45^a^	6.20 ± 0.28^a^	2.18 ± 0.18^b^	3.18 ± 0.05^a^	2.75 ± 0.13^a^	2.22 ± 0.15^b^	8.38 ± 0.44	7.47 ± 0.87	6.54 ± 1.62
∑n-3/∑n-6 PUFA	2.39 ± 0.26^a^	1.13 ± 0.09^b^	0.81 ± 0.03^b^	1.65 ± 0.36^a^	1.10 ± 0.19^ab^	0.48 ± 0.01^b^	1.09 ± 0.06^a^	0.62 ± 0.05^b^	0.45 ± 0.09^b^
∑n-3 LC-PUFA	6.06 ± 0.42^a^	5.06 ± 0.25^a^	1.23 ± 0.10^b^	2.91 ± 0.11^a^	2.09 ± 0.15^b^	1.01 ± 0.06^c^	7.84 ± 0.66^a^	2.89 ± 0.28^b^	0.79 ± 0.13^c^
Total fatty acids	21.58 ± 1.55^a^	26.36 ± 1.97^a^	14.43 ± 0.68^b^	14.29 ± 0.68	12.86 ± 0.71	14.63 ± 0.81	41.10 ± 1.63	39.99 ± 2.38	39.42 ± 3.76

^*^Values in the same row with no common superscript letters are significantly different (*P *< 0.05). Some fatty acids that were present in only minor or trace amounts or that were not detected, such as C22:0, C24:0, C14:1, C20:2n-6 and C20:3n-6, are not listed in the table.

^1^RFO: 100% Fish oil as lipid source (control) in rainbow trout.

^2^RFV: Vegetable oil blend (linseed oil: soya bean oil = 1:1) replacing 50% of fish oil in rainbow trout.

^3^RVO: 100% Vegetable oil blend as lipid source in rainbow trout.

^4^JFO: 100% Fish oil as lipid source (control) in Japanese seabass.

^5^JFV: Vegetable oil blend (linseed oil: soya bean oil = 1:1) replacing 50% of fish oil in Japanese seabass.

^6^JVO: 100% Vegetable oil blend as lipid source in Japanese seabass.

^7^LFO: 100% Fish oil as lipid source (control) in large yellow croaker.

^8^LFV: Vegetable oil blend (linseed oil: soya bean oil = 1:1) replacing 50% of fish oil in large yellow croaker.

^9^LVO: 100% Vegetable oil blend as lipid source in large yellow croaker.

^10^SFA: Saturated fatty acid.

^11^MUFA: Monounsaturated fatty acid.

^12^n-6 PUFA: n-6 poly-unsaturated fatty acid.

^13^n-3 PUFA: n-3 poly-unsaturated fatty acid.

**Table 3:** Liver fatty acid contents of three fish fed the experimental diets with vegetable oil instead of fish oil^1^ (mg/g, Mean ± SEM)*.

Fatty acid	RFO^1^	RFV^2^	RVO^3^	JFO^4^	JFV^5^	JVO^6^	LFO^7^	LFV^8^	LVO^9^
C14:0	0.49 ± 0.09^a^	0.27 ± 0.00^b^	0.20 ± 0.00^b^	1.42 ± 0.17	1.56 ± 0.03	1.70 ± 0.06	1.56 ± 0.19^a^	0.97 ± 0.02^b^	0.77 ± 0.07^b^
C16:0	8.66 ± 0.10^a^	6.61 ± 0.15^b^	6.70 ± 0.23^b^	8.57 ± 0.04^b^	8.42 ± 0.33^b^	10.36 ± 0.37^a^	17.43 ± 0.79^a^	18.61 ± 0.71^a^	14.16 ± 0.32^b^
C18:0	5.23 ± 0.11	5.45 ± 0.08	4.66 ± 0.32	4.25 ± 0.11^b^	4.65 ± 0.23^b^	6.81 ± 0.17^a^	10.44 ± 0.36^c^	14.35 ± 0.35^a^	12.59 ± 0.17^b^
∑SFA^10^	14.38 ± 0.19^a^	12.33 ± 0.13^b^	11.56 ± 0.30^b^	14.25 ± 0.32^b^	14.64 ± 0.56^b^	18.87 ± 0.48^a^	29.01 ± 1.01^b^	33.93 ± 0.61^a^	27.52 ± 0.22^b^
C16:1	1.63 ± 0.27^a^	0.62 ± 0.01^b^	0.38 ± 0.06^b^	2.33 ± 0.18	2.35 ± 0.07	2.30 ± 0.13	5.59 ± 0.33^a^	4.98 ± 0.19^ab^	3.85 ± 0.35^b^
C18:1	6.99 ± 0.45^b^	5.73 ± 0.25^b^	10.48 ± 0.71^a^	10.41 ± 0.31^c^	123.23 ± 0.15^b^	32.08 ± 0.61^a^	24.55 ± 0.89^c^	28.81 ± 0.34^b^	32.30 ± 0.09^a^
∑MUFA^11^	8.63 ± 0.40^b^	6.35 ± 0.24^c^	10.86 ± 0.77^a^	12.74 ± 0.49^c^	25.58 ± 0.22^b^	34.27 ± 0.68^a^	30.13 ± 1.10^b^	33.79 ± 0.53^a^	36.15 ± 0.27^a^
C18:2n-6	3.04 ± 0.45^b^	3.42 ± 0.11^b^	6.18 ± 0.21^a^	3.33 ± 0.29^b^	2.66 ± 0.35^b^	5.40 ± 0.39^a^	6.80 ± 0.40^c^	15.28 ± 0.29^b^	34.64 ± 0.26^a^
C20:4n-6	1.87 ± 0.16^b^	1.74 ± 0.34^b^	2.29 ± 0.41^a^	0.10 ± 0.00^a^	0.06 ± 0.01^b^	0.03 ± 0.00^c^	0.24 ± 0.02^a^	0.15 ± 0.01^b^	0.05 ± 0.02^c^
∑n-6 PUFA^12^	4.91 ± 0.54^b^	5.16 ± 0.15^b^	8.48 ± 0.23^a^	3.43 ± 0.29^b^	2.71 ± 0.36^b^	5.45 ± 0.38^a^	7.04 ± 0.39^c^	15.43 ± 0.30^b^	34.69 ± 0.27^a^
C18:3n-3	0.53 ± 0.09^b^	0.51 ± 0.02^b^	1.09 ± 0.03^a^	0.48 ± 0.08^b^	0.70 ± 0.08^ab^	0.84 ± 0.06^a^	1.49 ± 0.12^c^	4.36 ± 0.30^b^	6.74 ± 0.06^a^
C20:5n-3	1.29 ± 0.13^a^	0.72 ± 0.02^b^	0.78 ± 0.08^b^	0.20 ± 0.01^a^	0.06 ± 0.01^b^	0.03 ± 0.00^c^	1.34 ± 0.08^a^	0.71 ± 0.06^b^	0.14 ± 0.00^c^
C22:6n-3	18.60 ± 1.37^a^	13.38 ± 0.15^b^	11.89 ± 1.64^b^	0.65 ± 0.03^a^	0.16 ± 0.03^b^	0.07 ± 0.01^b^	2.64 ± 0.08^a^	1.21 ± 0.07^b^	0.27 ± 0.04^c^
∑n-3 PUFA^13^	20.42 ± 1.60^a^	14.61 ± 0.15^b^	13.76 ± 1.73^b^	1.33 ± 0.12^a^	1.34 ± 0.22^a^	0.94 ± 0.05^b^	5.47 ± 0.04^b^	6.27 ± 0.41^ab^	7.16 ± 0.01^a^
∑n-3/∑n-6 PUFA	4.21 ± 0.31^a^	2.84 ± 0.05^b^	1.62 ± 0.18^c^	0.39 ± 0.00^a^	0.34 ± 0.03^a^	0.17 ± 0.02^b^	0.78 ± 0.04^a^	0.41 ± 0.02^b^	0.21 ± 0.00^c^
∑n-3 LC-PUFA	19.89 ± 1.50^a^	14.10 ± 0.14^b^	12.67 ± 1.72^b^	0.85 ± 0.03^a^	0.22 ± 0.04^b^	0.10 ± 0.01^b^	3.98 ± 0.15^a^	1.92 ± 0.13^b^	0.42 ± 0.05^c^
Total fatty acids	43.35 ± 0.16	42.59 ± 1.61	45.28 ± 1.55	34.05 ± 1.37^c^	47.37 ± 1.30^b^	62.20 ± 0.91^a^	79.85 ± 3.15^c^	90.13 ± 3.51^ab^	104.86 ± 4.87^a^

^*^Values in the same row with no common superscript letters are significantly different (*P *< 0.05). Some fatty acids that were present in only minor or trace amounts or that were not detected, such as C22:0, C24:0, C14:1, C20:2n-6 and C20:3n-6, are not listed in the table.

^1^RFO: 100% Fish oil as lipid source (control) in rainbow trout.

^2^RFV: Vegetable oil blend (linseed oil: soya bean oil = 1:1) replacing 50% of fish oil in rainbow trout.

^3^RVO: 100% Vegetable oil blend as lipid source in rainbow trout.

^4^JFO: 100% Fish oil as lipid source (control) in Japanese seabass.

^5^JFV: Vegetable oil blend (linseed oil: soya bean oil = 1:1) replacing 50% of fish oil in Japanese seabass.

^6^JVO: 100% Vegetable oil blend as lipid source in Japanese seabass.

^7^LFO: 100% Fish oil as lipid source (control) in large yellow croaker.

^8^LFV: Vegetable oil blend (linseed oil: soya bean oil = 1:1) replacing 50% of fish oil in large yellow croaker.

^9^LVO: 100% Vegetable oil blend as lipid source in large yellow croaker.

^10^SFA: Saturated fatty acid.

^11^MUFA: Monounsaturated fatty acid.

^12^n-6 PUFA: n-6 poly-unsaturated fatty acid.

^13^n-3 PUFA: n-3 poly-unsaturated fatty acid.

**Table 4:** Formulation and proximate composition of the experimental diets (% of dry matter).

Ingredients	FO^1^	FV^2^	VO^3^
Defatted white fish meal^4^	15	15	15
Soybean meal	32	32	32
Casein^5^	11	11	11
Wheat meal	26	26	26
Mineral premix^6^	2	2	2
Vitamin premix^7^	2	2	2
Attractant^8^	0.3	0.3	0.3
Mould inhibitor^9^	0.1	0.1	0.1
Lecithin	2.6	2.6	2.6
Fish oil	9	4.5	0
Soybean oil	0	2.5	4.5
Linseed oil	0	2.5	4.5
Total	100	100	100
Proximate composition			
Crude protein	41.67	41.74	41.71
Crude lipid	12.85	12.70	12.76

^1^FO: 100% Fish oil as lipid source (control).

^2^FV: Vegetable oil blend (linseed oil: soya bean oil = 1:1) replacing 50% of fish oil.

^3^VO: 100% Vegetable oil blend as lipid source.

^4^Defatted fish meal: 72.1% Crude protein and 1.4% crude lipid; white fish meal were defatted with ethanol (fish meal: ethanol = 1:2, w:v) at 37 °C three times.

^5^Casein: 88% Crude protein and 1.3% crude lipid, Alfa Aesar, Avocado Research Chemicals Ltd, UK.

^6^Mineral premix (mg or g kg^-1^ diet): CuSO_4_·5H_2_O 10 mg; Na_2_SeO_3_ (1%) 25 mg; ZnSO_4_·H_2_O, 50 mg; CoC_l2_·6H_2_O (1%) 50 mg; MnSO_4_·H_2_O 60 mg; FeSO_4_·H_2_O 80 mg Ca (IO_3_)_2_ 180 mg; MgSO_4_·7H_2_O 1200 mg; zeolite 18.35 g.

^7^Vitamin premix (mg or g kg^−1^ diet): Vitamin D 5 mg; vitamin K 10 mg vitamin B12 10 mg vitamin B6 20 mg; folic acid 20 mg; vitamin B1 25 mg; vitamin A 32 mg; vitamin B2 45 mg; pantothenic acid 60 mg; biotin 60 mg; niacin acid 200 mg; α-tocopherol 240 mg; inositol 800 mg; ascorbic acid 2000 mg; microcrystalline cellulose 16.47 g.

^8^Phagostimulant: Glycine: betaine = 1:3.

^9^Preservative: Fumarate: calcium propionate = 1:1.

**Table 5:** The contents of various fatty acids in the experimental diets (mg/g)^1^.

Fatty acid	FO	FV	VO
C14:0	0.76	0.42	0.10
C16:0	4.51	3.96	3.13
C18:0	1.63	1.72	1.71
∑SFA^2^	6.90	6.10	4.94
C16:1	1.08	0.53	0.06
C18:1	3.59	4.58	5.47
∑MUFA^3^	4.67	5.11	5.53
C18:2n-6	4.35	8.85	12.66
C20:4n-6	0.12	0.07	0.04
∑n-6 PUFA^4^	4.47	8.93	12.70
C18:3n-3	0.43	3.32	6.98
C20:5n-3	1.25	0.62	0.06
C22:6n-3	1.85	0.88	0.08
∑n-3 PUFA^5^	3.53	4.82	7.12
∑n-3/∑n-6 PUFA	0.79	0.54	0.56
∑n-3 LC-PUFA	3.10	1.49	0.14
Total fatty acids	21.18	26.82	31.09

^1^Some fatty acids that were present in only minor or trace amounts or that were not detected, such as C22:0, C24:0, C14:1, C20:2n-6 and C20:3n-6, are not listed in the table.

^2^SFA: Saturated fatty acid.

^3^MUFA: Monounsaturated fatty acid.

^4^n-6 PUFA: n-6 poly-unsaturated fatty acid.

^5^n-3 PUFA: n-3 poly-unsaturated fatty acid.

**Table 6:** Primers used in real-time PCR.

Species	Gene	Primer	Sequence (5′-3′)
Rainbow trout	SREBP-1	RS-F	CAAGCTGCCCATCAACCGTA
		RS-R	GGCCACCAGGTCTTTAAGCTC
	PPAR-α1	RP1-F	TCCCCCAGTCCATCGACGGTGACA
		RP1-R	GAGAACTCCTCCAGCCCTGCAGCT
	PPAR-α2	RP2-F	CAGTCGAGTAACTGCTCTGGCT
		RP2-R	ACAGGCGTGAACTCCATAGTGGT
	FADS2	RFADS2-F	GTCCGTGCTTTGTGTGAGAA
		RFADS2-R	TCAGAGACCCGACAACATCA
	β-actin	R-β-actin-F	TCCTTCCTCGGTATGGAGTCT
		R-β-actin-R	TTACGGATGTCCACGTCACAC
Japanese seabass	SREBP-1	JS-F	TGCTATCGGTTCTAACATGGCTAC
		JS-R	AGTGCTCAACAGTCAGATACAGTC
	PPAR-α1	JP1-F	AAGACCAGCACCCCTCCTTTCGT
		JP1-R	CCGAACTTCTGCCTCCCTGTCCT
	PPAR-α2	JP2-F	TTCCAGCTGGCAGAGAGGACGC
		JP2-R	CACCCCACAGCCGGAACCACCT
	FADS2	JFADS2-F	CCGTCGCGACTGGGTGGATATG
		JFADS2-R	CAGTCCCGGTGCTTCTCGTGG
	β-actin	J-β-actin-F	CAACTGGGATGACATGGAGAAG
		J-β-actin-R	TTGGCTTTGGGGTTCAGG
Large yellow croaker	SREBP-1	YS-F	TCTCCTTGCAGTCTGAGCCAAC
		YS-R	TCAGCCCTTGGATATGAGCCT
	PPAR-α1	YP-F	AAGTGCCTCTCTGTGGGAATGT
		YP-R	TCACCTCTTTCTCCACCATCT
	FADS2	YFADS2-F	TTCGCTTCCTCTGCTGCTATG
		YFADS2-R	CCAGTCACGGTGCTTCTCG
	β-actin	Y-β-actin-F	CTACGAGGGTTATGCCCTGCC
		Y-β-actin-R	TGAAGGAGTAACCGCGCTCTGT
