# Supplementary material for: Regulation of FADS2 transcription by SREBP-1 and PPAR-α influences LC-PUFA biosynthesis in fish
Source: Sci Rep. 2017 Jan 9;7:40024. doi: 10.1038/srep40024 (PMC5220380; doi:10.1038/srep40024)
Supplement: Supplementary Information [file srep40024-s1.pdf]

Regulation of FADS2 transcription by SREBP-1 and PPAR-α influences LC-PUFA biosynthesis in fish

Xiaojing Dong [a], Peng Tan[a], Zuonan Cai[a], Hanlin Xu[a], Jingqi Li[a], Wei Ren[a], Houguo Xu[a], Rantao Zuo[a], Jianfeng Zhou[b], Kangsen Mai[a], Qinghui Ai[a, c,] *

[a] Key Laboratory of Aquaculture Nutrition and Feed (Ministry of Agriculture) and Key Laboratory of Mariculture (Ministry of Education), Ocean University of China, 5 Yushan Road, Qingdao, Shandong 266003, People's Republic of China

[b] Key Laboratory of Marine Drugs, Ministry of Education, Ocean University of China, 5 Yushan Road, Qingdao, Shandong 266003, People's Republic of China

[c]Laboratory for Marine Fisheries and Aquaculture, Qingdao National Laboratory for Marine Science and Technology, Qingdao, Shandong 266003, People's Republic of China

*Corresponding author:

Qinghui Ai

Tel: +86 532 82031943

Fax: +86 532 82031943

E-mail address: qhai@ouc.edu.cn

Figure legends

Fig. S1. Comparison of the N-terminal protein sequences of SREBP-1 between fishes and human.

Fig. S2. Phylogenetic relationship between the amino acid sequences of SREBP-1 from vertebrate and invertebrate.

Fig. S3. Comparison of the deduced amino acid sequences of PPAR-α between fishes and human. The DNA-binding domain and ligand-binding domain is solid and dash underlined respectively.

Tables

Table S1. Primers used in PCR for FADS2 promoter cloning and characterization.

**Species**	**Primer**	**Sequence (5’-3’)**
	Rsp1	GCAAAGTGACTGATGACCCTGAT
Rainbow trout	Rsp2	TGACCAACCACTTGTCGCTTC
	Rsp3	CTCGCTTGACTCCGTCTGTTG
	Jsp1	CGTGGCATCCTCTCCAGAATAGTG
Japenese seabass	Jsp2	ACTGGTCGTTCCTGCTGCGGT
	Jsp3	AGCTGACCCCCGCCTCCCAT
	Ysp1	TCCTCTCCAGCATAGTGTCCGAT
Large yellow croaker	Ysp2	GACCATCCACTGATCATTCCTGT’
	Ysp3	CAGGTGTAAACGCCATCATCTCG

Table S2. Primers used in PCR for SREBP-1 and PPAR-α cloning and characterization.

Species	Gene	Primer	Sequence (5’-3’)	Sequence information
Two Fishes	SREBP-1	SREBP-1F	TCTGGAGRCAYCGCAARCAGGC;	RT primer
		SREBP-1R	GTGGGRCTGGCCCCNGCCATC	RT primer
	PPAR-α	PPAR-αF	TGGTCGGATGCCACAGGCGGAGAAGC	RT primer
		PPAR-αR	GAACTGGAAYTTGGGCTCCATC	RT primer
Rainbow trout	SREBP-1	R-S-3F1	ATGGTGGTCGTCGGTTGCCGTGGT	3’RACE
		R-S-3F2	CGCCGAGCACCTGCCCCGTAGTCT	3’RACE
	PPAR-α2	R-P-3F1	GCAGAGCAGACCCTCGTTGCCAAGAT	3’RACE
		R-P-3F2	GAGGCTGAGGTCCGCATCTTCCACTG	3’RACE
	SREBP-1	R-S-5R1	GCTATCTTTGGGGGTGCTGCGGACG	5’RACE
		R-S-5R2	ATGTGAACCGCAGACAGGTGGCTAC	5’RACE
	PPAR-α2	R-P-5R1	AGTGGAAGATGCGGACCTCAGCCTC	5’RACE
		R-P-5R2	CCACCATCTTGGCAACGAGGGTCTG	5’RACE
Large yellow croaker	SREBP-1	Y-S-3F1	AGACTGTATCTGACTGTTGAGCACTT	3’RACE
		Y-S-3F2	AAGGTCGTCCAGTTGCTCTTGTGTGAT	3’RACE
	SREBP-1	Y-S-5R1	AGACGGGCTGAGAGTGGGTTGTGGC	5’RACE
		Y-S-5R2	GGAGGAACTACGGGTTATGGGAGGGC	5’RACE

Table S3. Primers used in recombinant vector construction.

**Species**	**Gene**	**Primer**	**Sequence (5'-3')**	**Sequence information**
Rainbow trout	FADS2	PGL-RF-F	TAGGTACCAGACGGGGCTGTCCTAT	reporter plasmids
		PGL-RF-R	CCAAGCTTCATCCTCGGTTCTCTC	reporter plasmids
	SREBP-1	PCS-RS-F	CCGGAATTCATGAACTTGTCTT	expression plasmids
		PCS-RS-R	CCGCTCGAGCTAGGCAGAGGTGAC	expression plasmids
	PPAR-α1	PCS-RP1-F	CCTGAATTCATGGCGAGCCACT	expression plasmids
		PCS-RP1-R	CTCCTCGAGTCAGTACATGTCCCTATAGATCTCCTG	expression plasmids
	PPAR-α2	PCS-PR2-F	CCTGAATTCATGGCGAGCCACT	expression plasmids
		PCS-PR2-R	CTCCTCGAGTCAGTACATGTCCCTATAGATCTCCTG	expression plasmids
Japenese seabass	FADS2	PGL-JF-F	GGGGTACCAAGATTACTTATAGGTCT	reporter plasmids
		PGL-JF-R	CCAAGCTTAATCCTCAGCGCTG	reporter plasmids
	SREBP-1	PCS-JS-F	CCGGAATTCATGAACAGCCTGTCTT	expression plasmids
		PCS-JS-R	CCGCTCGAGCTAGCTGTTGGTGACG	expression plasmids
	PPAR-α1	PCS-JP1-F	CCGGAATTCATGGTCGACATGGAGAG	expression plasmids
		PCS-JP1-R	CCGCTCGAGTCAGTACATGTCTCTG	expression plasmids
	PPAR-α2	PCS-JP2-F	CCGGAATTCATGGCAGGGGATCTCTTTAG	expression plasmids

		PCS-JP2-R	CCGCTCGAGTCAGTACATGTCCCTGTAT	expression plasmids
	FADS2	PGL-YF-F	GCCGGTACCTGTGTATAATGGAGC	reporter plasmids
		PGL-YF-R	CCAAGCTTAATCCTCACTGCTGTC	reporter plasmids
Large yellow croaker	SREBP-1	PCS-YS-F	CCGGAATTCATGAACAGCCTGTC	expression plasmids
		PCS-YS-R	CCGCTCGAGCTAGCTGTTGGTGAC	expression plasmids
	PPAR-α	PCS-YP-F	CCGGAATTCATGGCAGGGATTTCT	expression plasmids
		PCS-YP-R	CCGCTCGAGTCAGTACATGTCCCTGTAT	expression plasmids

Figures

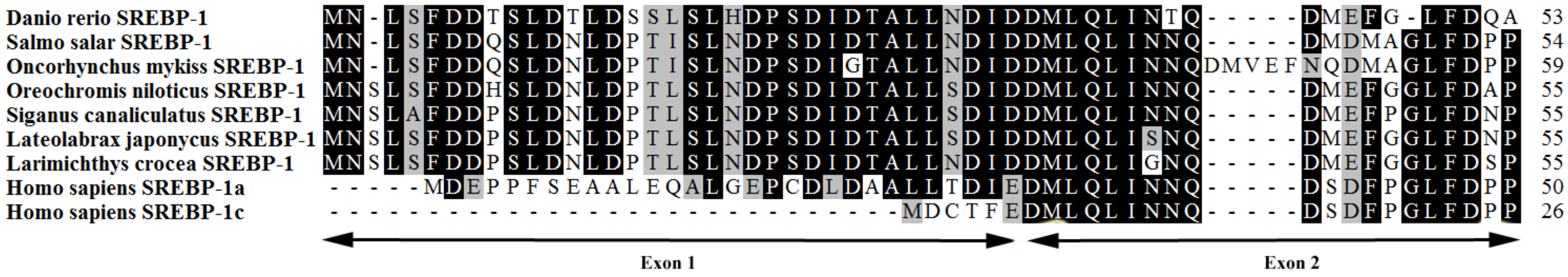

Figure S1. Comparison of the N-terminal protein sequences of SREBP-1 between fishes and human.

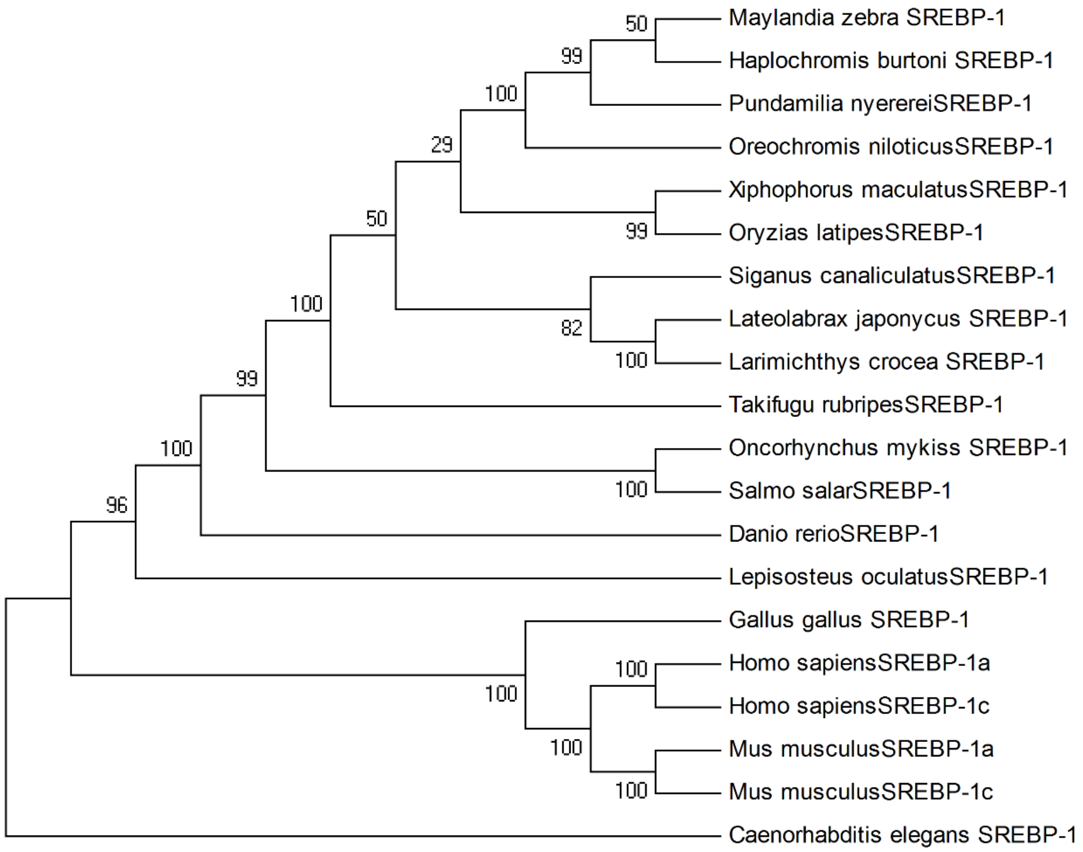

Figure S2. Phylogenetic relationship between the amino acid sequences of SREBP-1 from vertebrate and invertebrate.

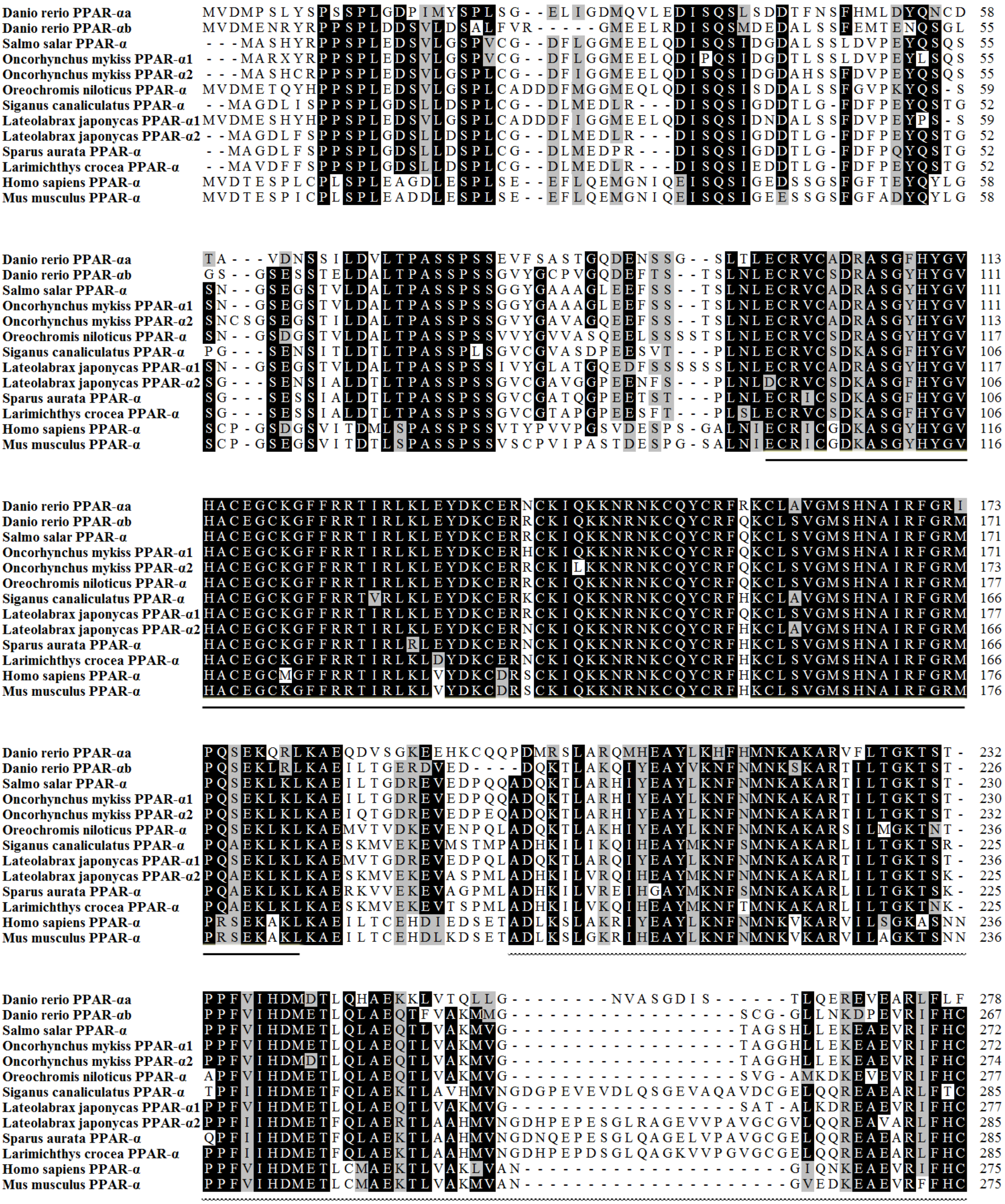

Danio rerio PPAR-αa MVDMPSLYSPSSPLGDPIMYSPLSG--ELIGDMQVLEDISQSLSDDTFNSFHMLDYQNCD 58
Danio rerio PPAR-αb MVDMENRYRPPSPLDDSVLDSALFVR----GMEELRDISQSMDEDALSSFEMTENQSGL 55
Salmo salar PPAR-α ---MASHYRPPSPLEDSVLGSPVCG--DFLGGMEELQDISQSIDGDALSSLDVPEYQSQS 55
Oncorhynchus mykiss PPAR-α1 ---MARXYRPPSPLEDSVLGSPVCG--DFLGGMEELQDIPQSIDGDTLSSLDVPEYLSQS 55
Oncorhynchus mykiss PPAR-α2 ---MASHCRPPSPLEDSVLGSPLCG--DFIGGMEELQDISQSIDGDAHSSFDVPEYQSQS 55
Oreochromis niloticus PPAR-α MVDMETQYHPPSPLEDSVLGSPLCADDDFMGGMEQLQDISQSISDDALSSFGVPKYQS-S 59
Siganus canaliculatus PPAR-α --MAGDLISPPSPLGDSLLDSPLCG--DLMEDLR---DISQSIGDDTLG-FDFPEYQSTG 52
Lateolabrax japonycas PPAR-α1 MVDMESHYHPPSPLEDSVLGSPLCADDDFIGGMEELQDISQSIDNDALSSFDVPEYPS-S 59
Lateolabrax japonycas PPAR-α2 --MAGDLFSPPSPLGDSLLDSPLCG--DLMEDLR---DISQSIGDDTLG-FDFPEYQSTG 52
Sparus aurata PPAR-α --MAGDLFSPPSPLGDSLLDSPLCG--DLMEDPR---DISQSIGDDTLG-FDFPQYQSTG 52
Larimichthys crocea PPAR-α --MAVDFFSPPSPLGDSLLDSPLCG--DLMEDLR---DISQSIEDDTLG-FDFPEYQSTG 52
Homo sapiens PPAR-α MVDTESPLCPLSPLEAGDLESPLSE--EFLQEMGNIQEISQSIGEDSSGSFGFTEYQYLG 58
Mus musculus PPAR-α MVDTESPICPLSPLEADDLESPLSE--EFLQEMGNIQEISQSIGEESSGSFGFADYQYLG 58

Danio rerio PPAR-αa TA---VDNSSILDVLTPASSPSSEVFSASTGQDENSSG--SLTLECRVCADRASGFHYGV 113
Danio rerio PPAR-αb GS--GSESSTELDALTPASSPSSGVYGCPVGQDEFTS--TSLNLECRVCSDRASGYHYGV 111
Salmo salar PPAR-α SN--GSEGSTVLDALTPASSPSSGGYGAAAGLEEFSS--TSLNLECRVCADRASGYHYGV 111
Oncorhynchus mykiss PPAR-α1 SN--GSEGSTVLDALTPASSPSSGGYGAAAGLEEFSS--TSLNLECRVCADRASGYHYGV 111
Oncorhynchus mykiss PPAR-α2 SNCSGSEGSTILDALTPASSPSSGVYGAVAGQEEFSS--TSLNLECRVCADRASGYHYGV 113
Oreochromis niloticus PPAR-α SN--GSDGSTVLDALTPASSPSSVVYGVVASQEELSSSTSLNLECRVCSDRASGYHYGV 117
Siganus canaliculatus PPAR-α PG---SENSITLDTLTPASSPLSGVCGVASDPEESVT---PLNLECRVCSDKASGFHYGV 106
Lateolabrax japonycas PPAR-α1 SN--GSEGSTVLDALTPASSPSSIVYGLATGQEDFSSSSSLNLECRVCADRASGYHYGV 117
Lateolabrax japonycas PPAR-α2 SG---SENSIALDTLTPASSPSSGVCGAVGGPEENFS---PLNLDCRVCSDKASGFHYGV 106
Sparus aurata PPAR-α SG---SESSIALDTLTPASSPSSGVCGATQGPEETST---PLNLECRICSDKASGFHYGV 106
Larimichthys crocea PPAR-α SG---SESSIALDTLTPASSPSSGVCGTAPGPEESFT---PLSLECRVCSDKASGFHYGV 106
Homo sapiens PPAR-α SCP-GSDGSVITDMLSPASSPSSVTYPVVPGSVDESPS-GALNIECRICGDKASGYHYGV 116
Mus musculus PPAR-α SCP-GSEGSVITDTLSPASSPSSVSCPVIPASTDESPG-SALNIECRICGDKASGYHYGV 116

Danio rerio PPAR-αa HACEGCKGFFRRTIRLKLEYDKCERNCKIQKKNRNKCQYCRFRKCLAVGMSHNAIRFGRI 173
Danio rerio PPAR-αb HACEGCKGFFRRTIRLKLEYDKCERRCKIQKKNRNKCQYCRFQKCLSVGMSHNAIRFGRM 171
Salmo salar PPAR-α HACEGCKGFFRRTIRLKLEYDKCERRCKIQKKNRNKCQYCRFQKCLSVGMSHNAIRFGRM 171
Oncorhynchus mykiss PPAR-α1 HACEGCKGFFRRTIRLKLEYDKCERHCKIQKKNRNKCQYCRFQKCLSVGMSHNAIRFGRM 171
Oncorhynchus mykiss PPAR-α2 HACEGCKGFFRRTIRLKLEYDKCERRCKILKKNRNKCQYCRFQKCLSVGMSHNAIRFGRM 173
Oreochromis niloticus PPAR-α HACEGCKGFFRRTIRLKLEYDKCERRCKIQKKNRNKCQYCRFQKCLSVGMSHNAIRFGRM 177
Siganus canaliculatus PPAR-α HACEGCKGFFRRTVRLKLEYDKCERKCKIQKKNRNKCQYCRFHKCLAVGMSHNAIRFGRM 166
Lateolabrax japonycas PPAR-α1 HACEGCKGFFRRTIRLKLEYDKCERNCKIQKKNRNKCQYCRFQKCLSVGMSHNAIRFGRM 177
Lateolabrax japonycas PPAR-α2 HACEGCKGFFRRTIRLKLEYDKCERNCKIQKKNRNKCQYCRFHKCLAVGMSHNAIRFGRM 166
Sparus aurata PPAR-α HACEGCKGFFRRTIRLRLEYDKCERNCKIQKKNRNKCQYCRFHKCLSVGMSHNAIRFGRM 166
Larimichthys crocea PPAR-α HACEGCKGFFRRTIRLKLDYDKCERNCKIQKKNRNKCQYCRFHKCLSVGMSHNAIRFGRM 166
Homo sapiens PPAR-α HACEGCMGFFRRTIRLKLVYDKCDRSCKIQKKNRNKCQYCRFHKCLSVGMSHNAIRFGRM 176
Mus musculus PPAR-α HACEGCKGFFRRTIRLKLVYDKCDRSCKIQKKNRNKCQYCRFHKCLSVGMSHNAIRFGRM 176

Danio rerio PPAR-αa PQSEKQRLKAEQDVSGKEEHKCQQPDMRSLARQMHEAYLKHFHMNKAKARVFLTGKTST- 232
Danio rerio PPAR-αb PQSEKLRLKAEILTGERDVED----DQKTLAKQIYEAYVKNFNMNKSKARTILTGKTST- 226
Salmo salar PPAR-α PQSEKLKLKAEILTGDREVEDPQQADQKTLARHIYEAYLKNFNMNKAKARTILTGKTST- 230
Oncorhynchus mykiss PPAR-α1 PQSEKLKLKAEILTGDREVEDPQQADQKTLARHIYEAYLKNFNMNKAKARTILTGKTST- 230
Oncorhynchus mykiss PPAR-α2 PQSEKLKLKAEIQTGDREVEDPEQADQKTLARHIYEAYLKNFNMNKAKARTILTGKTST- 232
Oreochromis niloticus PPAR-α PQSEKLKLKAEMVTVDKEVENPQLADQKTLAKHIYEAYLKNFNMNKAKARSILMGKTNT- 236
Siganus canaliculatus PPAR-α PQAEKLKLKAESKMVEKEVMSTMPADHKILIKQIHEAYMKNFSMNKAKARLILTGKTSR- 225
Lateolabrax japonycas PPAR-α1 PQSEKLKLKAEMVTGDREVEDPQLADQKTLARQIYEAYLKNFNMNKAKARTILTGKTST- 236
Lateolabrax japonycas PPAR-α2 PQAEKLKLKAESKMVEKEVASPMLADHKILVRQIHEAYMKNFNMNKAKARLILTGKTSK- 225
Sparus aurata PPAR-α PQAEKLKLKAERKVVEKEVAGPMLADHKILVREIHGAYMKNFSMNKAKARLILTGKTSK- 225
Larimichthys crocea PPAR-α PQAEKLKLKAESKMVEKEVTSPMLADHKILVKQIHEAYMKNFTMNKAKARLILTGKTNK- 225
Homo sapiens PPAR-α PRSEKAKLKAEILTCEHDIEDSETADLKSLAKRIYEAYLKNFNMNKVKARVILSGKASNN 236
Mus musculus PPAR-α PRSEKAKLKAEILTCEHDLKDSETADLKSLGKRIHEAYLKNFNMNKVKARVILAGKTSNN 236

Danio rerio PPAR-αa PPFVIHDMDTLQHAEKKLVTQLLG--------NVASGDIS------TLQEREVEARLFLF 278
Danio rerio PPAR-αb PPFVIHDMETLQLAEQTFVAKMMG------------------SCG-GLLNKDPEVRIFHC 267
Salmo salar PPAR-α PPFVIHDMETLQLAEQTLVAKMVG------------------TAGSHLLEKEAEVRIFHC 272
Oncorhynchus mykiss PPAR-α1 PPFVIHDMETLQLAEQTLVAKMVG------------------TAGGHLLEKEAEVRIFHC 272
Oncorhynchus mykiss PPAR-α2 PPFVIHDMDTLQLAEQTLVAKMVG------------------TAGGHLLEKEAEVRIFHC 274
Oreochromis niloticus PPAR-α APFVIHDMETLQLAEQTLVAKMVG------------------SVG-AMKDKEVEVRIFHC 277
Siganus canaliculatus PPAR-α TPFIIHDMETFQLAEKTLAVHMVNGDGPEVEVDLQSGEVAQAVDCGELQQREAEARLFTC 285
Lateolabrax japonycas PPAR-α1 PPFVIHDMETLQLAEQTLVAKMVG------------------SAT-ALKDREAEVRIFHC 277
Lateolabrax japonycas PPAR-α2 PPFIIHDMETFQLAERTLAAHMVNGDHPEPESGLRAGEVVPAVGCGVLQQREAVARLFHC 285
Sparus aurata PPAR-α QPFIIHDMETFQLAEKTLAAHMVNGDNQEPESGLQAGELVPAVGCGELQQREAEARLFHC 285
Larimichthys crocea PPAR-α PPFIIHDMETFQLAEKTLAAHMVNGDHPEPDSGLQAGKVVPGVGCGELQQREAEARLFHC 285
Homo sapiens PPAR-α PPFVIHDMETLCMAEKTLVAKLVAN---------------------GIQNKEAEVRIFHC 275
Mus musculus PPAR-α PPFVIHDMETLCMAEKTLVAKMVAN---------------------GVEDKEAEVRFFHC 275

```
Danio rerio PPAR-αa              CQYASVATVTELTEYAKAVPGFADLDLNDQVTLLKYGVYEALFTLLASCMNKDGLLVAQG  338
Danio rerio PPAR-αb              CQCTSVETVTELTEFAKSVPGFSNLDLNDQVTLLKYGVHEALFAMLASCMNKDGLLVAYG  327
Salmo salar PPAR-α               CQCTSVETVTELTEFAKSVPGFSSLDLNDQVTLLKYGVYEALFALLASCMNKDGLLVAYG  332
Oncorhynchus mykiss PPAR-α1      CQCTSVETVTELTEFAKSVPGFSSLDLNDQVTLLKYGVYEALFALLASCMNKDGLLVAYG  332
Oncorhynchus mykiss PPAR-α2      CQCTSVETVTELTEFAKSVPGFSSLDLNDQVTLLKYGVYEALFALLASCMNKDGLLVAYG  334
Oreochromis niloticus PPAR-α     CQCTSVETVTELTEFAKSVPGFSNLDLNDQVTLLKYGVYEAIFAMLASSMNKDGLLVAYG  337
Siganus canaliculatus PPAR-α     CQSTSVETVTELTEFAKAVPGFQSLDLNDQVTLLKYGVYEAFFTLLASCMNKDGLLVARG  345
Lateolabrax japonycas PPAR-α1    CQCTSVETVTELTEFAKSVPGFSNLDLNDQVTLLKYGVYEALFAMLASSMNKDGLLVAYG  337
Lateolabrax japonycas PPAR-α2    CQSTSVETVTELTEFAKAVPGFQSLDLNDQVTLLKYGVYEALFTLLASCMNKDGLLVARG  345
Sparus aurata PPAR-α             CQSTSVETVTELTEFAKAVPGFQSLDLNDQVTLLKYGVYEALFTLLASCMNKDGLLVARG  345
Larimichthys crocea PPAR-α       CQSTSVETVTELTEFAKAVPGFQSLDLNDQVTLLKYGVYEALFTLLASCMNKDGLLVARG  345
Homo sapiens PPAR-α              CQCTSVETVTELTEFAKAIPGFANLDLNDQVTLLKYGVYEAIFAMLSSVMNKDGMLVAYG  335
Mus musculus PPAR-α              CQCMSVETVTELTEFAKAIPGFANLDLNDQVTLLKYGVYEAIFTMLSSLMNKDGMLIAYG  335

Danio rerio PPAR-αa              GGFITREFLKSLRKPFSDMMEPKFQFAMKFNALELDDSDLALFVAAIICCGDRPGLSNVP  398
Danio rerio PPAR-αb              SGFITREFLKSLRRPFSDMMEPKFQFAMKFNSLELDDSDLALFVAAIICCGDRPGLVNVP  387
Salmo salar PPAR-α               SGFITREFLKSLRRPFSDMMEPKFQFAMKFNGLELDDSDLALFVAAIICCGDRPGLVNVT  392
Oncorhynchus mykiss PPAR-α1      SGFITREFLKSLRRPFSDMMEPKFQFAMKFNGLELDDSDLALFVAAIICCGDRPGLVNVT  392
Oncorhynchus mykiss PPAR-α2      SGFITREFLKSLRRPFSDMMEPKFQFAMKFNGLELDDSDLALFVAAIICCGDRPGLVNVG  394
Oreochromis niloticus PPAR-α     SGFITREFLKSLRRPFSDMMEPKFQFAMKFNALELDDSDLALFVAAIICCGDRPGLVNVA  397
Siganus canaliculatus PPAR-α     GGFITREFLKSLRRPFGDMMEPKFQFASRFNSLELDDSDLALFVAAIICCGDRPGLVDVP  405
Lateolabrax japonycas PPAR-α1    SGFITREFLKSLRQPFSEMMEPKFQFAMKFNALELDDSDLALFVAAIICCGDRPGLVNVA  397
Lateolabrax japonycas PPAR-α2    GGFITREFLKSLRRPFSDMMEPKFQFATRFNSLELDDSDLALFVAAIICCGDRPGLVDVP  405
Sparus aurata PPAR-α             GGFITREFLKSLRRPFSDMMEPKFQFATRFNSLELDDSDLALFVAAIICCGDRPGLVDVP  405
Larimichthys crocea PPAR-α       GGFITREFLKSLRRPFSDMMEPKFQFATRFNSLELDDSDLALFVAAIICCGDRPGLVDVP  405
Homo sapiens PPAR-α              NGFITREFLKSLRKPFCDIMEPKFDFAMKFNALELDDSDISLFVAAIICCGDRPGLLNVG  395
Mus musculus PPAR-α              NGFITREFLKNLRKPFCDIMEPKFDFAMKFNALELDDSDISLFVAAIICCGDRPGLLNIG  395

Danio rerio PPAR-αa              QIERIQESVIHSLRLHLTSNHPDNSLLFPKLLQKLADLRQLVTEHAELVQEINKTEDAS-  457
Danio rerio PPAR-αb              HIERMQESIVNVLHLHLKSNHPDHGFLFPKLLQKLVDLRQLVTEHAQLIQEIKKTEDTS-  446
Salmo salar PPAR-α               HIECMQENIVQVLQLHLLANHPDDTFLFPNLLQKLADLRQLVTEHAQLVQEIKKTEDTS-  451
Oncorhynchus mykiss PPAR-α1      HIECMQENIVQVLQLHLLANHPDDTFLFPNLLQKLADLRQLVTEHAQLVQEIKKTEDTS-  451
Oncorhynchus mykiss PPAR-α2      HIERMQENIVQVLRLHLLANHPDDTFLFPKLLQKLSDLRQLVTEHAQLVQEIKKTEDMS-  453
Oreochromis niloticus PPAR-α     HIEAMQESIVQILQLHLLANHPDDTFLFPRLLQKLADLRQLVTEHAQLVQEIKKTEDTS-  456
Siganus canaliculatus PPAR-α     LVEQLQESIVQVLRLHLLANHPDDNFLFPRLLQKLADLRELVTEHAQLVQEIKTMEDTS-  464
Lateolabrax japonycas PPAR-α1    HIERMQDSIVQVLQLHLLSNHPDDAFLFPRLLQKLADLRQLVTEHAQLVQEIKKTEDTS-  456
Lateolabrax japonycas PPAR-α2    LVEQLQESIVQALRLHLLANHPDDNFLFPRLLQKLADLRELVTEHAQLVQEIKTTEDTS-  464
Sparus aurata PPAR-α             LVEQLQESIVQVLRLHLLANHPDDTFLFPRLLQKLADLRELVTEHAQLVQEIKTTEDTS-  464
Larimichthys crocea PPAR-α       LVEQLQESIVQALRLHLLANHPDDHFLFPRLLQKLADLRELVTEHAQLVQEIKTTEDTS-  464
Homo sapiens PPAR-α              HIEKMQEGIVHVLRLHLQSNHPDDIFLFPKLLQKMADLRQLVTEHAQLAQIIKKTESDAA  455
Mus musculus PPAR-α              YIEKLQEGIVHVLKLHLQSNHPDDTFLFPKLLQKMVDLRQLVTEHAQLVQVIKKTESD--  453

Danio rerio PPAR-αa              LHPLLQEIYRDMY  470
Danio rerio PPAR-αb              LHPLLQEIYRDMY  459
Salmo salar PPAR-α               LHPLLQEIYRDMY  464
Oncorhynchus mykiss PPAR-α1      LHPLLQEIYRDMY  464
Oncorhynchus mykiss PPAR-α2      LHPLLQEIYRDMY  466
Oreochromis niloticus PPAR-α     LHPLLQEIYRDMY  469
Siganus canaliculatus PPAR-α     LHPLLQEIYRDMY  477
Lateolabrax japonycas PPAR-α1    LHPLLQEIYRDMY  469
Lateolabrax japonycas PPAR-α2    LHPLLQEIYRDMY  477
Sparus aurata PPAR-α             LHPLLQEIYRDMY  477
Larimichthys crocea PPAR-α       LHPLLQEIYRDMY  477
Homo sapiens PPAR-α              LHPLLQEIYRDMY  468
Mus musculus PPAR-α              -------------  453
```

Fig. S3. Comparison of the deduced amino acid sequences of PPAR-α between fishes and human.
